# Neutropenia and Neutropenic Complications in ABVD Chemotherapy for Hodgkin Lymphoma

**DOI:** 10.1155/2011/656013

**Published:** 2011-05-02

**Authors:** Bhanu Vakkalanka, Brian K. Link

**Affiliations:** Department of Internal Medicine, Holden Comprehensive Cancer Center, University of Iowa, Iowa City, IA 52242, USA

## Abstract

A combination of Adriamycin (a.k.a. Doxorubicin), Bleomycin, Vinblastine, and Dacarbazine (ABVD) is the most commonly used chemotherapy regime for Hodgkin lymphoma. This highly effective treatment is associated with a significant risk of neutropenia. Various strategies are adopted to counter this commonly encountered problem, including dose modification, use of colony stimulating factors, and prophylactic or therapeutic use of antibiotics. Data to support these approaches is somewhat controversial, and in keeping with the paucity of definitive evidence, there is a wide disparity in the management of neutropenia in patients receiving ABVD chemotherapy. This paper summarizes the evidence for managing ABVD-related neutropenia during the treatment of Hodgkin lymphoma.

## 1. Introduction

Hodgkin Lymphoma (HL) accounts for 10–15% of all lymphomas in the western countries. It typically shows a bimodal age distribution and accounts for 15% of all cancers in the population aged 15–24 years [1]. The incidence of HL, based on data from the United States and European registries ranges from 2.3 to 3.2 per 100,000 men and 1.3 to 2.5 per 100,000 women. The estimated 5-year survival of all HL patients is about 86%, but the survival drops to 53% in patients older than 65 years of age (SEER database) [2]. 

HL is classified into classical HL and lymphocyte predominant type, based on differences in morphology, genotype, phenotype, and clinical behavior. Classical HL is again subdivided into four distinct histological types: nodular sclerosis, mixed cellularity, lymphocyte predominant and lymphocyte depleted [3]. Patients present at various clinical stages with or without B symptoms, as defined by the Cotswold staging system [4]. 

## 2. Advanced Hodgkin Treatment Overview

Combined chemotherapy and radiation is the most effective treatment approach for early stage (I-II) HL. Chemotherapy with involved field radiation therapy (IFRT) is shown to be superior to radiation therapy alone in a large EORTC clinical trial [5]. A metaanalysis of twenty-three clinical trials suggests that use of this combined modality treatment results in better tumor control and overall survival compared to chemotherapy alone [6]. Most trials have shown that two to four cycles of chemotherapy with IFRT is adequate treatment for early stage HL [5, 7, 8]. 

Advanced HL (defined as presentation with tumor bulk > 10 cm, the presence of B symptoms, and/or stage III/IV disease) is associated with a 30–40% failure rate following anthracycline-based chemotherapy [9]. Six to eight cycles of chemotherapy are considered optimal in the initial treatment of advanced HL, based on several clinical trials [10–14]. 

## 3. ABVD Chemotherapy

ABVD is the most commonly used treatment for both early and advanced HL in the USA, and several other western nations. Two hundred forty-six of 283 (87%) patients with HL from a University of Iowa/Mayo Clinic prospective observational database received ABVD as initial chemotherapy in 2003–present [15]. This regimen is also favored in densely populated but less prosperous countries where the drugs are available at costs fairly lower than those in the United States. Several clinical trials have proven that ABVD is highly effective and has a favorable toxicity profile in comparison to other chemotherapy regimens used in advanced HL. A CALGB randomized trial compared three regimens of chemotherapy against each other in patients with advanced HL: (1) MOPP (Mechlorethamine, vincristine, procarbazine, and prednisone) alone—the then standard of treatment—, given for 6 to 8 cycles (2) MOPP alternating with ABVD for 12 cycles (3) ABVD alone for 6 to 8 cycles. ABVD was shown to have a 5-year failure-free survival (FFS) of 61% and an overall survival of 73%, and was as effective as the MOPP/ABVD alternating regimen and superior to MOPP alone. It was also found to be less myelotoxic than either of the MOPP containing regimens and has become the standard of treatment for HL and the reference arm for trials exploring other novel chemotherapy combinations [10]. 

ABVD was also compared with two multidrug regimens (MDRs) in the UK LY09 trial [16] which randomized patients to ABVD or two different MDRs. At a median followup of 52 months, no differences in event-free or overall survival were noted between the treatment groups, but substantially more grade 3-4 adverse effects were noted in the patients receiving MDRs. The Italian GISL study [17] compared ABVD, BEACOPP (bleomycin, etoposide, doxorubicin, cyclophosphamide, vincristine, procarbazine, and prednisone), and CEC (cyclophosphamide, lomustine, vindesine, melphalan, prednisone, epidoxirubicin, vincristine, procarbazine, vinblastine, and bleomycin) with each other. There was no difference in the overall survival between the arms, but the BEACOPP regime showed a statistically significant improvement in 5-year PFS, compared to ABVD (81% versus 65%, *P* = .038). However, BEACOPP was associated with considerably higher rates of grade 3-4 anemia (16% versus 5%), neutropenia (54% versus 34%) and infections (14% versus 2%). 

ABVD was also compared against Stanford V, an abbreviated (12 week) polychemotherapy regimen developed to minimize the late toxicity of chemotherapy for advanced HL. A three arm Italian study evaluating ABVD, modified Stanford V, and MOPPEBVCAD showed that OS was superior in the ABVD group compared to the modified Stanford [14] regime (90% versus 82% *P* = .04). However, the study was criticized for the nonuniform and delayed use of radiation therapy in the modified Stanford V arm. A UK NCRI randomized trial of 520 patients, available as an abstract only, also compared Stanford V to ABVD chemotherapy [18]. At a median followup of 4 years, a preplanned interim analysis shows no difference in PFS and OS between the two groups. Pulmonary toxicity was higher in the ABVD group while other toxicities were more common with Stanford V. Data from these various clinical trials provide the basis for the acceptance of ABVD as the most preferred first line treatment choice for HL. 

Despite being the least toxic of the chemotherapy regimes available to treat HL, ABVD is still associated with significant adverse effects. The typical duration of the treatment cycle is 28 days, and the standard doses of the drugs are provided in Table 1. 

The commonest grade 3-4 acute adverse effects of this regime, based on the Italian GISL study, include neutropenia (34%), alopecia (31%), nausea/vomiting (13%), anemia (5%), thrombocytopenia (3%), infections (2%), constipation (2%), and mucositis (1%) [17]. Long-term toxicities of ABVD chemotherapy include pulmonary toxicity (8%), cardiac dysfunction (3%), and secondary malignancies (4%) [13].

## 4. ABVD and Neutropenia

Neutropenia is a common complication of ABVD chemotherapy, but related complications including febrile neutropenia (FN), neutropenic sepsis, and death are much less frequent. The frequency of grade 3 or 4 neutropenia—defined by National Cancer Institute (NCI) common terminology criteria for adverse events (CTCAE) as an absolute neutrophil count (ANC) of <1.0–0.5 × 10^9^/L and <0.5 × 10^9^/L, respectively—in randomized trials involving ABVD chemotherapy ranges from 10 and 66% [13, 19, 20]. The wide range of the occurrence of this problem possibly reflects the varied number of treatment cycles administered in these trials. The incidence of febrile neutropenia or neutropenic sepsis is not specified in these prospective studies. The incidence of grade 3 infection (with or without neutropenia) in a CALGB study was 2% [10]. A further study from the Memorial Sloan Kettering Cancer Center reported an 8% rate of hospitalization due to neutropenia in patients receiving ABVD chemotherapy [21]. 

The degree and duration of neutropenia is directly related to the incidence and severity of febrile neutropenia and infections [22]. Hence higher grades of neutropenia (grade 3 and 4) often lead to consideration of several strategies to prevent associated complications and also to maintain high dose intensity. There is a wide variability in the approach to this problem, but the main strategies adopted to achieve this goal include (1) dose delay/modifications (2) growth factor (GF) support, (3) prophylactic antibacterial therapy. 

### 4.1. Dose Modifications/Delays

Dose modification was widely adopted in the earlier studies comparing MOPP regimens with ABVD [10, 23]. In the study reported by Canellos et al., a 75% dose reduction of Dacarbazine and Vinblastine is implemented for a leucocyte count of 2.5 × 10^9^/L to 3.5 × 10^9^/L at the time of the next dose. All chemotherapy drugs were withheld if the WBC count is <2.5 × 10^9^/L. Despite a significant number of patients in the MOPP regimens requiring dose alterations or delays, more than 80% of patients in the ABVD arm received >85% of the intended dose of doxorubicin. Similarly, >80% compliance to the intended drug delivery is achieved with ABVD chemotherapy in other clinical trials, suggesting that the treatment is generally well tolerated. 

Studies evaluating dose intensity in MOPP-based chemotherapy for HL have shown that administering full doses without treatment delays results in improved disease-free and overall survival [24, 25]. However, the precise level of dose intensity required to achieve these better outcomes was not clearly defined [26]. It was also shown in a cohort of elderly patients, with a median age of 72 yrs, receiving ABVD-based chemotherapy, that those who received >65% of the intended chemotherapy dose intensity had better cause specific survival (CSS) and overall survival (OS) [27]. 

Some of the limitations of the above data are that these studies are based on older chemotherapy regimens and the exact dose intensity of ABVD chemotherapy required to improve survival in HL is unknown. The Goldie-Coldman hypothesis suggests that outcomes of cancer treatment are improved with delivery of intended chemotherapy doses on time [28]. A strong argument can be made, that it is even more imperative to maintain a high dose intensity in this chemosensitive malignancy [29]. The practice of routine dose modifications/delays based on isolated neutropenia abrogates the end point of delivering optimal doses on time. However, this strategy is still an integral part of several clinical trial designs in HL and we believe that this approach needs reevaluation in the future.

### 4.2. ABVD and Growth Factor Support

While most ABVD-based clinical trials initiated prior to the introduction of GFs relied on dose alterations as the prime strategy to counter neutropenia, subsequent studies relied on GFs as the preferred way to maintain dose intensity and to prevent neutropenia-related complications [14, 16, 20, 21]. Both granulocyte colony stimulating factor (G-CSF) and granulocyte macrophage colony stimulating factor (GM-CSF) have been used to manage neutropenia in cancer chemotherapy, but neither of them is found to be superior to the other [30]. A Cochrane collaboration review evaluated the role of colony stimulating factors (both G-CSF and GM-CSF) in the treatment of malignant lymphomas [31]. A total of 2607 patients from 13 randomized control trials of patients with both HL and Non-Hodgkin lymphoma (NHL) were included in the review. The relative risk of severe neutropenia, febrile neutropenia, and infections was lower, but freedom from treatment failure (FFTF) and OS were not improved with institution of GFs. 

Several retrospective series suggest that growth factors are not required to maintain high dose intensity in the treatment of HL. A study from the University of Iowa reported that FN developed only in 8 of 81 patients who received a total of 894 treatments (half cycles) [32]. GF support was used at the discretion of the clinician in 58 of 187 (37%) treatments associated with grade 3 or 4 neutropenia on the day of treatment, and dose modifications were implemented in 29 (15.5%) of these treatments. Eight of a total of 9 episodes of FN developed in patients with an ANC of >1.0 × 10^9^/L, none of whom received GFs. Of the 158 treatments administered without dose modification, one episode of FN occurred in the 58 treatments accompanied by G-CSF and none in the 100 treatments given without G-CSF support. This study concludes that neutropenic fever is uncommon (1%) and unrelated to neutrophil count on the day of ABVD treatment. In another study reported from the Northwestern University, 61 out of 84 analyzed patients proceeded with full dose ABVD therapy without dose delay or G-CSF support, irrespective of the ANC on the day of treatment [26]. Of note, monocytosis was used as an indicator for imminent neutrophil recovery and was found to be consistently elevated above the normal range, across all treatment cycles. Febrile neutropenia was noted in only 3 of 682 treatments (0.44%), despite a high number of treatments (58%) delivered with an ANC of ≤1 × 10^9^/L on the day of ABVD treatment. Fifty-nine of these patients reached 99% target dose intensity without GF support, and the cycle duration and dose intensity in this group were not statistically different to a comparison group of twenty-three patients who received routine empiric G-CSF. Of note, nearly all patients received prophylactic trimethoprim-sulfamethoxazole and fluconazole which might have had a protective effect on systemic infections. Five-year event-free and overall survival rates in the patients not receiving G-CSF were 92.9% and 97.4%, respectively, and compared favorably with those receiving G-CSF. 

Two further retrospective studies from the UK also conclude that a preponderant majority of patients completed their ABVD chemotherapy as planned, without GFs. In the study by Boleti and Mead, 36 of 38 patients (95%) were able to complete their intended ABVD chemotherapy without any increased risk of infective episodes [33]. About 79% of the patients had one or more episodes of grade 3 or 4 neutropenia, but the incidence of febrile neutropenia was only 0.57% of all injection days (two injection days per cycle). Neither GFs nor dose modifications were used to manage ABVD-related neutropenia in this study. In the second study by Nangalia et al., a total of 263 ABVD treatments (131.5 cycles) were delivered to 24 patients [34]. None of these patients received GFs and all patients received full dose of treatment irrespective of their ANC, provided no other cytopenias or toxicities were found on the day of treatment. Forty-seven and eighteen percent of these patients had grade 3 and grade 4 neutropenia, respectively, but the incidence of FN was only 0.76%. Ninety-six percent of the patients received the planned treatment without any dose reductions for hematological toxicity, suggesting that it is possible to safely and effectively administer ABVD chemotherapy irrespective of the neutrophil count on the day of treatment, and that neutropenia is a poor surrogate for FN. The results of these various studies are summarized in Table 2. Growth factor-related side effects, albeit minor, need to be seriously considered while initiating therapy with these agents. A report from Martin et al. suggests an increased incidence of Bleomycin pulmonary toxicity (BPT) in patients receiving G-CSF compared to those without G-CSF (26% versus 9% *P* = .014) [35]. Those patients with BPT have lower median 5-year survival compared to unaffected patients (63% versus 90%). Also, usage of GFs is associated with substantial costs, the estimates of which vary widely. In the study by Evens et al., the net savings per patient on the pharmaceutical costs of G-CSF use are estimated to be approximately $1800 [26]. 

The guidelines from the American Society of Clinical Oncology (ASCO) and the European Organization for Research and Treatment of Cancer (EORTC) recommend the use of colony stimulating factors (CSF) as primary prophylaxis in patients who have a >20% risk of developing FN from chemotherapy [36, 37]. The ASCO guidelines further recommend GFs for secondary prophylaxis in the event of a prior neutropenic complication. These and other data referred above, show that the incidence of FN in patients treated with ABVD is far less than 20%, suggesting that primary prophylaxis with GFs is not indicated in these patients. The added benefits of cost savings and minimizing toxicity make a strong argument for the omission of colony stimulating factors as routine prophylaxis for isolated neutropenia in the delivery of ABVD.

## 5. ABVD and Prophylactic Use of Antibiotics

Another strategy to prevent FN and neutropenia-related infectious complications, is the administration of prophylactic antibacterial therapy. There are no randomized controlled trials (RCTs) addressing the role of prophylactic antibiotics, exclusively in patients receiving ABVD chemotherapy. However, several RCTs that include large numbers of HD patients reported the benefits of such a therapeutic intervention in preventing neutropenic complications. 

In the GEMIMA study, Bucaneve et al. analyzed 760 patients receiving chemotherapy for solid tumors, lymphomas, and acute leukemias, who were at increased risk of developing prolonged neutropenia (more than 7 days) [38]. These patients were stratified into two groups, those with acute leukemia versus solid tumors or lymphoma, and were randomized to receive either oral levofloxacin or placebo from the start of chemotherapy until the resolution of neutropenia. A total of 212 patients with either Non Hodgkin or Hodgkin lymphoma were recruited into this study, but details of the exact number of patients with HL and their chemotherapy regimens were not provided. In both groups, levofloxacin, compared to placebo, was associated with a significantly lower rate of neutropenic fever, microbiologically documented infections and bacteremias. However, no survival benefit was seen in patients receiving antibiotic therapy. 

In the SIGNIFICANT study by Cullen et al., 1565 patients with various solid tumors and lymphomas on chemotherapy were randomized to receive levofloxacin or placebo during the anticipated neutropenic period [39]. A total of 59 patients (3.1%) had HL, and the majority of them received ABVD chemotherapy. The primary end point was incidence of fever, clinically documented as a temperature of >38°C. 

There was a significantly lower incidence of febrile episodes in those receiving levofloxacin compared to placebo (10.8% versus 15.2%; *P* = .01). A reduction of 38 days of hospitalization was achieved for every 100 patients treated with prophylactic levofloxacin. Almost half of the deaths reported in the study occurred in the first month of chemotherapy, and no survival benefit was seen with antibiotic usage. No data was presented regarding the antibacterial resistance of the infecting organisms. Notably, 38% of the patients treated with levofloxacin developed fevers outside the expected time of neutropenia, raising questions of the predictability of neutrophil nadir counts and timing of antibiotic prophylaxis. 

Gafter-Gvili et al. reported a metaanalysis of 95 clinical trials between 1973 and 2004, in an attempt to address the role of prophylactic antibiotic therapy in patients receiving chemotherapy for various malignancies [40]. This study suggested that there is a survival benefit associated with administration of antibiotics, particularly with fluoroquinolones (relative risk, 0.52 (CI, 0.35 to 0.77)). A large limitation of the study is that majority of the patients in these clinical trials had hematological malignancies with competing causes for an increased risk of infection, and data on all cause mortality was missing in 10 out of 50 trials comparing antibiotic prophylaxis with no intervention. The RCTs by Bucaneve et al. and Cullen et al., which had a large number of patients with HL, were not included in this meta-analysis. In a subsequent smaller meta-analyses of four trials comparing flouroquinolones to placebo in patients with solid tumors or lymphoma (including the GEMIMA and SIGNIFICANT trials), the authors conclude that flouroquinolones, when used as primary prophylaxis, at least for the first cycle of chemotherapy, confer a survival benefit [41]. 

The proportion of patients with HL in these studies is small, and full details of the chemotherapy received by them are not available. Retrospective data suggest that the incidence of neutropenic complications in patients receiving ABVD chemotherapy is low (<1%). Hence, we believe that there is no role for routine administration of prophylactic antibiotics for these patients. 

## 6. ABVD-Related Neutropenia and HIV Patients 

Patients with human immunodeficiency virus (HIV) infection and acquired immune deficiency syndrome (AIDS) are at an increased risk of developing HL. Though not an AIDS-defining illness, the incidence of HL is high in these patients and even higher in the highly active antiretroviral therapy (HAART) era [42]. “B” symptoms are more frequently seen in patients with HIV-related HL, who often present at advanced stages (stages III and IV) and carry a poorer prognosis compared to HL patients without HIV infection [43, 44]. Chemotherapy in this population has to be undertaken with special care, in view of the immunocompromised state of these patients. 

No randomized data are available to confirm the best chemotherapy regimen in these patients. In a prospective nonrandomized trial, 21 patients with HIV-related HL received ABVD chemotherapy with primary G-CSF prophylaxis, but no HAART therapy. Despite G-CSF prophylaxis, 52% of patients developed ≥ grade 3 toxicity. Six patients (29%) developed opportunistic infections while on chemotherapy, and 2 patients died from infectious complications within 3 months of therapy. The median OS was 18 months, far less than expected with ABVD therapy for HL in the general population. Subsequent trials showed that administering HAART along with chemotherapy is associated with better outcomes [45]. In a retrospective multicenter study, 62 HIV-related HL patients were treated with ABVD and HAART, and G-CSF was administered in 20% of patients, at the discretion of the participating institutions. Six patients (9.7%) died during the induction phase of chemotherapy, largely from infectious complications. Five-year EFS and OS were 71% and 76%, respectively, lower than that expected in HL patients without HIV infection [46]. 

Clearly, the incorporation of HAART along with ABVD chemotherapy improves survival and reduces the infection-related complications in patients with HIV-related HL. There are no data to prove the role of primary prophylaxis with GFs even in this population, but given their higher susceptibility to infections, it would be reasonable to consider GF support and or prophylactic antibiotics in this high-risk group.

## 7. Conclusions

Hodgkins lymphoma is exquisitely responsive to ABVD chemotherapy, which results in a high cure rate. In order to achieve maximum benefit from this chemotherapy, it is important to maintain optimal dose intensity. Severe neutropenia is a well-recognized complication of this regimen, which in theory could lead to an increased risk of morbidity and mortality from infectious complications. There is a wide variation in the several strategies adapted by physicians to balance these conflicting priorities of maintaining dose intensity while preventing neutropenia and related complications. These strategies include dose modifications and/or GF support and/or prophylactic antibiotics. Available evidence suggests that although severe neutropenia is a common occurrence with ABVD therapy, FN and other related complications are rare in this setting. Patients can safely proceed with ABVD chemotherapy in the presence of severe neutropenia, without other therapeutic interventions. This approach potentially avoids pulmonary toxicity and reduces cost without loss of therapeutic efficacy. Several ongoing multicenter and international trials in Hodgkin lymphoma currently utilize “full dose” ABVD without modifications for neutropenia. These prospective trials should confirm or refute the safety findings of the retrospective analyses described above. Special patient groups such as HIV patients would need other concomitant measures such as HAART therapy to reduce infectious complications. Though not optimally studied, a strong case can be made to administer GFs with or without prophylactic antibiotics in this special population with a high rate of infectious complications.

## Figures and Tables

**Table 1:** ABVD doses and schedule.

Drug	Dosage and Route	Days of Treatment
Doxorubicin	25 mg/m^2^ I.V	1 and 15
Bleomycin	10 mg/m^2^ I.V	1 and 15
Vinblastine	6 mg/m^2^ I.V	1 and 15
Dacarbazine	375 mg/m^2^ I.V	1 and 15

**Table 2:** Retrospective studies showing poor correlation between neutropenia and neutropenic complications.

Author	Number of analyzed patients without G-CSF	Grade 4 neutropenia (%) patients/treatments^1^	% of febrile neutropenia^2^	% of growth factor supported treatments	DM^3^ due to grade 3 or 4 neutropenia
Chand et al.	81	43/7	1.0	6.5	15.5% of all treatments
Evens et al.	59	26/NA^4^	0.44	0	None
Boleti and Mead	28	37/NA	0.57	0	None
Nangalia et al.	24	NA/18	0.76	0	None

1. Treatments are half cycles, that is, two treatments per cycle.

2. Incidence of febrile neutropenia as percentage of overall treatments (half cycles).

3. DM: dose modification.

4. NA: not available.
